# Completely extradural intraspinal arteriovenous malformation in the lumbar spine: a case report

**DOI:** 10.1186/1752-1947-8-216

**Published:** 2014-06-23

**Authors:** Saravanabavaan Suntharalingam, Adrian Ringelstein, Michael Forsting, Ulrich Sure, Johannes van de Nes, Oliver Gembruch

**Affiliations:** 1 Department of Radiology and Neuroradiology, University Hospital Essen, Hufelandstr. 55, 45122 Essen, Germany; 2 Department of Neurosurgery, University Hospital Essen, Hufelandstr. 55, 45122 Essen, Germany; 3 Department of Neuropathology, University Hospital Essen, Hufelandstr. 55, 45122 Essen, Germany

**Keywords:** Arteriovenous malformation, Intraspinal extradural lesion, Spinal vascular malformation

## Abstract

**Introduction:**

Spinal vascular malformations can be classified in arteriovenous malformations, cavernomas, and capillary telangiectasias. Arteriovenous malformations are the most common spinal vascular anomaly and may be located intra- and/or perimedullary. According to their nidus type and hemodynamic flow patterns, they can be differentiated into fistulous, glomerular and juvenile categories. In our case, a hyperintense extradural mass was misinterpreted as a neurinoma. The histological analysis revealed typical signs of an arteriovenous malformation.

**Case presentation:**

A 57-year-old Caucasian woman presented with back pain and hypesthesia in digiti two to four of her left foot. Magnetic resonance imaging showed a long-segment intraspinal extradural soft-tissue mass in the left L4 - S1 paravertebral region with homogeneous enhancement of contrast medium. Due to another similar lesion in the lower ankle and additional cutaneous manifestations, the suspected diagnosis was a systemic disease with neurinomas (e.g. Recklinghausen’s disease). To clear up the origin and type of this lesion exploratory surgery with a hemilaminectomy of L5 was performed. This showed abnormally arterialized, dilated, and tortuous vessels. After complete resection, the intra-operative impression of an arteriovenous malformation was confirmed by a neuropathologist.

**Conclusions:**

Completely extradural intraspinal arteriovenous malformations in the lumbar spine are extremely rare. In magnetic resonance imaging they are often misinterpreted as a tumor. Arteriovenous malformations can cause compression and venous congestion, or mask symptoms like a spinal disk herniation.

In cases presenting with these symptoms and magnetic resonance imaging findings, an extradural intraspinal arteriovenous malformation should be considered as a possible diagnosis. Pre-operative angiography or magnetic resonance imaging angiography can be used to verify the diagnosis.

## Introduction

Arteriovenous malformations (AVMs) are the most common spinal vascular anomaly and can be located intra- and/or perimedullary. According to their nidus type and hemodynamic flow patterns they can be differentiated into fistulous, glomerular and juvenile categories [1].

In our case, an extradural mass with contrast enhancement in the lumbar spine was misinterpreted as a neurinoma. However, histological examination revealed the typical characteristics of an AVM.

## Case presentation

**Figure 1:**
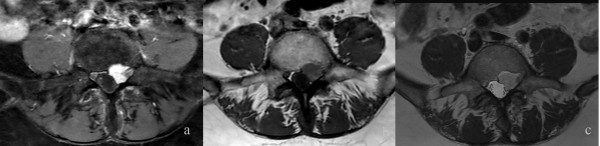
**Magnetic resonance imaging findings.** The T2-TSE **(b)** and the T1-TSE **(c)** sequence reveals an intraspinal, extradural soft-tissue mass in the left L4 - S1 paravertebral region. It is compressing the nerve roots of L4 and L5. The T1 SPIR **(a)** sequence with contrast medium shows the homogenous enhancement of this lesion.

**Figure 2:**
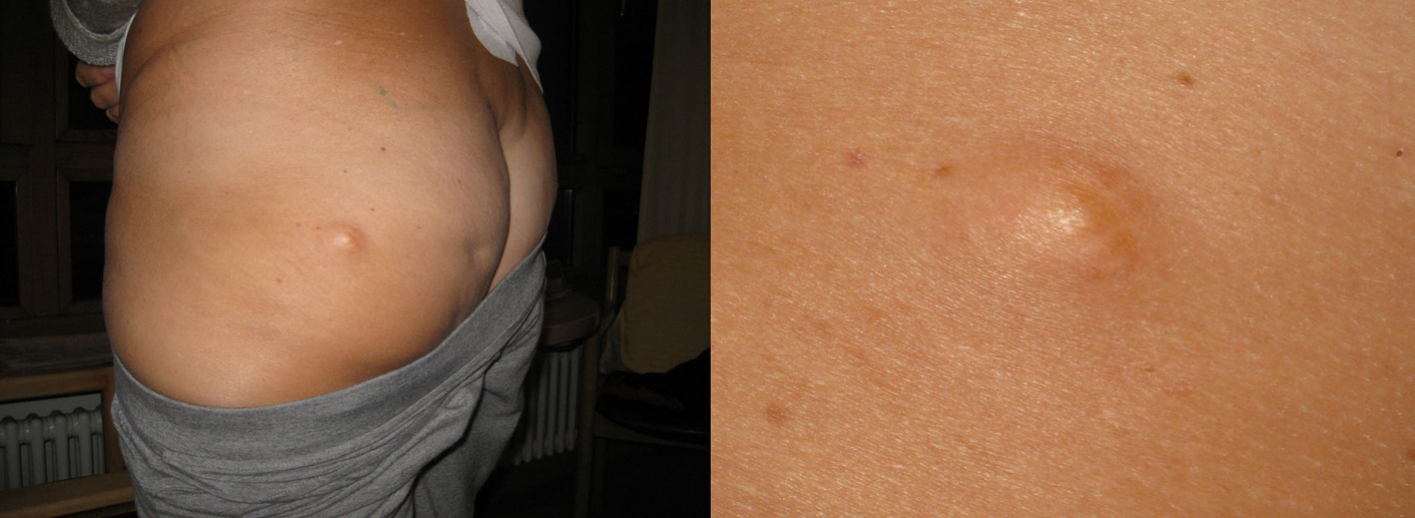
Cutaneous lesion which could have been a neurofibroma.

**Figure 3:**
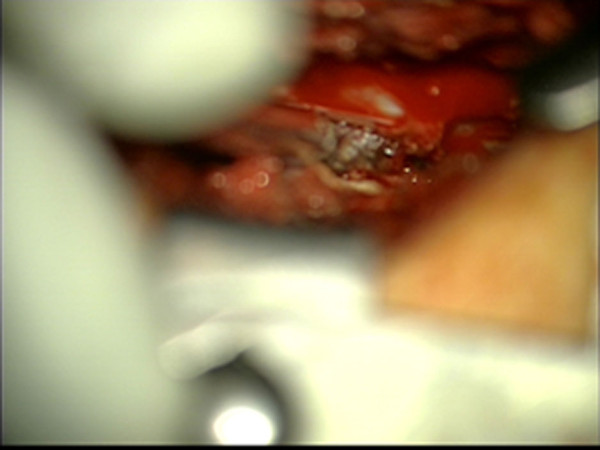
**Intraoperative finding.** Abnormal arterialized, dilated, and tortuous vessels were found while the hemilaminectomy was being performed.

**Figure 4:**
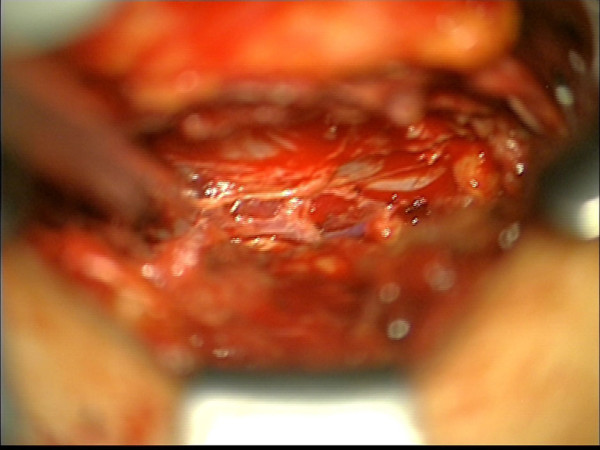
**Intraoperative finding.** Abnormal arterialized, dilated, and tortuous vessels were found while the hemilaminectomy was being performed.

**Figure 5:**
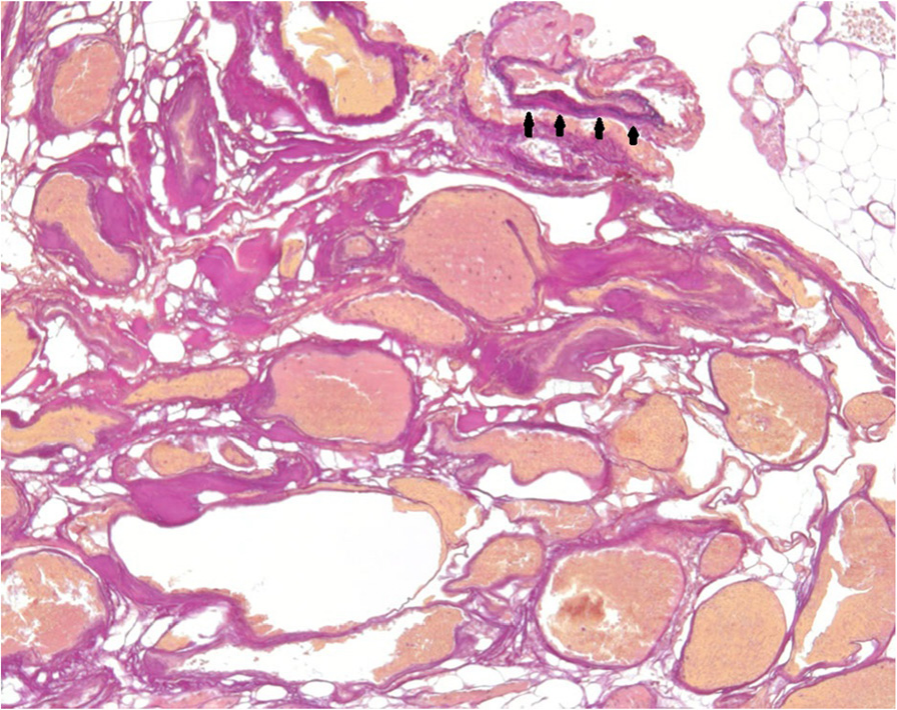
**A photomicrograph of the arteriovenous malformation, composed of variably sized blood vessels with variable wall thickness and some intervening fibrofatty tissue.** The arterial differentiation of some blood vessels is highlighted by the presence of a lamina elastica interna, stained black in the Elastica-van-Gieson stain (arrow heads). Original magnification × 100.

A 57-year-old Caucasian woman presented to our hospital with a tumor in her lower ankle. She reported hypesthesia in digiti two to four of her left foot. She also presented with temporary back pain.A magnetic resonance imaging (MRI) scan of her lumbar spine revealed a long-segment intraspinal extradural soft-tissue mass in the left L4 - S1 paravertebral region with homogeneous contrast enhancement. The mass was compressing the nerve roots of L4 and L5 and causing bone destruction of the fifth lumbar vertebra (Figure 1). It was at this stage that the diagnosis of a neurinoma was made.An MRI of her left foot revealed a space-occupying lesion at the lower ankle with homogeneous enhancement of contrast medium similar to that of the spinal lesion. The second lesion was also suspected to be a neurinoma. In addition, there was also a cutaneous lesion which could have been a neurofibroma (Figure 2). Overall, a systemic disease like neurofibromatosis (Recklinghausen’s disease) was considered as a diagnosis.To confirm the origin and type of the intraspinal lesion, exploratory surgery was performed over a left-sided hemilaminectomy of L5 and a partial hemilaminectomy of L4 and S1, followed by preparation towards the dura. The location of the lesion was completely extradural along the nerve roots L5 and S1. Abnormal arterialized, dilated, and tortuous vessels suggestive of an AVM were found (Figures 3 and 4). A hemostasis procedure and subsequent removal of the lesion followed, using bipolar coagulation. A further decompression of the nerve roots L5 and S1 was then performed.The intra-operative impression of an AVM was confirmed by a neuropathologist. A microscopic examination revealed a conglomerate of irregularly spaced blood vessels of variable size, composed of blood vessels with arterial, venous, and intermediate differentiation with an uneven thickness (Figure 5).

No neurological deficit occurred after the operation. She had an uneventful recovery and was discharged seven days later. During the clinical follow-up three months post-surgery, the pain in her lower back had subsided, but the hypesthesia in digiti two to four of her left foot had remained. An MRI scan showed a regular recovery with no signs of a residuum.

## Discussion

Spinal AVMs are often located intra- and/or perimedullarily. Roughly 25% of all spinal vascular shunts are performed on AVMs. The different types usually become symptomatic in early adulthood. Medullary damage can be caused by venous congestion and hemorrhage. The space-occupying effect is rarely the reason for the symptoms [1].

Only a few cases of lumbar intraspinal extradural AVMs have been published [2,3]. The previous imaging in these cases demonstrated no evidence of an AVM. In the case report of Marshman *et al.*[2] only prominent epidural veins were noted. Han *et al.*[3] reported that the image findings led to a working diagnosis of a plexiform neurofibroma. In both cases the suspected diagnosis had to be revised. In contrast to our case the lesions were not initially removed and aspinal angiography was later performed which revealed extradural AVMs.

The typical appearance of spinal cord AVMs in an MRI scan is as a conglomerate of dilated vessels that are demonstrated on T2-weighted sequences as flow voids, in T1-weighted sequences (depending on their flow velocity and direction) they present mixed hyper/hypointense tubular structures. Often these lesions have dense contrast enhancement. They can cause damage by acute intramedullary or subarachnoidal hemorrhage [4]. If intraparenchymal hemorrhages are present, depending on the time elapsed between bleeding and imaging, the image may become even more complicated due to varying signal intensities [1].

In our case this contrast-enhancing lesion has been misinterpreted as a neurinoma, as its signal characteristics and affinity to contrast medium appeared similar to neurinomas [5]. Additionally there were hints of a systemic disease with neurinomas (e.g. Recklinghausen’s disease).

After analysis of all the sequences, other differential diagnoses (for example a solitary lymphoma or a metastasis) were possible because of a similar appearance in the MRI scan, but an AVM was not considered at all.

Despite the unexpected intra-operative finding, this lesion could be completely removed by the neurosurgeon without foreseeable complications. In cases such as these, the German Society of Neurosurgery recommends in its guidelines a pre-operative angiography. This is necessary to define the anatomy of the involved vessels and to evaluate the hemodynamics. This in turn, makes it possible to classify the AVM exactly, to decrease the operative risk, and to improve the post-operative outcome. According to these guidelines, a non-invasive magnetic resonance angiography can also be used to detect a spinal AVM.

## Conclusions

A completely extradural intraspinal AVM in the lumbar spine is extremely rare. In MRI scans they are often misinterpreted as a tumor. They can cause compression and venous congestion, and cause the same symptoms as a spinal disk herniation.

In cases presenting with these symptoms and MRI findings, an extradural intraspinal AVM should be considered as a possible diagnosis. A pre-operative angiography or magnetic resonance angiography can be used to verify the diagnosis.

## Consent

Written informed consent was obtained from the patient for publication of this case report and any accompanying images. A copy of the written consent is available for review by the Editor-in-Chief of this journal.

## Abbreviations

AVM: arteriovenous malformation; MRI: magnetic resonance imaging; SPIR: Spectral Presaturation with Inversion Recovery; TSE: Turbo Spin Echo.

## Competing interests

The authors declare that they have no competing interests.

## Authors’ contributions

SS was a major contributor in writing the manuscript. SS, AR and MF analyzed the images. US performed the neurosurgical procedure. OG was part of the neurosurgical team and was also a major contributor in writing the manuscript. JN performed the pathological examination. All authors read and approved the final manuscript.
